# An Improved Biometrics-Based Remote User Authentication Scheme with User Anonymity

**DOI:** 10.1155/2013/491289

**Published:** 2013-11-21

**Authors:** Muhammad Khurram Khan, Saru Kumari

**Affiliations:** ^1^King Saud University, P.O. Box 92144, Riyadh 11653, Saudi Arabia; ^2^Department of Mathematics, Agra College, Agra, Dr. B. R. A. University, Agra, Uttar Pradesh 282002, India

## Abstract

The authors review the biometrics-based user authentication scheme proposed by An in 2012. The authors show that there exist loopholes in the scheme which are detrimental for its security. Therefore the authors propose an improved scheme eradicating the flaws of An's scheme. Then a detailed security analysis of the proposed scheme is presented followed by its efficiency comparison. The proposed scheme not only withstands security problems found in An's scheme but also provides some extra features with mere addition of only two hash operations. The proposed scheme allows user to freely change his password and also provides user anonymity with untraceability.

## 1. Introduction

In the last two decades, digital authentication has originated as a preferred method to authenticate remote users over insecure networks. After the first proposal of user authentication scheme by Lamport [1], considerable amount of research has been conducted in this field of which schemes [1–25] are few examples. In due course of time user authentication schemes underwent many changes. Initial schemes were based only on password [1–4], then schemes were based on smart card and password [5–13], and reliability of biometrics authentication over traditional password-based authentication gave rise to biometrics-based user authentication schemes [14–20]. 

In 2010, Li and Hwang [19] proposed a biometrics-based user authentication scheme. In 2011, Das [26] examined Li-Hwang's scheme and observed problems in login and authentication phase, in password change phase, and in biometrics verification mechanism of the scheme. Das depicted that user's smart card does not validate the inputted password during login phase which leads to useless computations in login and authentication phase. Owing to the same reason, Das further showed that the scheme suffers from incorrect password updating problem. Thus, Das proposed an improvement [26] of Li-Hwang's scheme and claimed their scheme to be free from problems observed in Li-Hwang's scheme. According to Das, their scheme [26] also provides mutual authentication. In 2012, An [27] pointed out that Das's scheme [26] deviates from the author's claim since an adversary can mount impersonation attacks and password guessing attack once he gets a chance to extract values from the smart card of the legal user. Thereby An [27] proposed an enhanced scheme to eradicate the flaws of Das's scheme.

In this paper, we review An's biometrics-based user authentication scheme. We show that An's scheme is vulnerable to the security problems to which Das's scheme is susceptible like online and offline password guessing attacks, user and server impersonation attacks, lack of mutual authentication, and lack of user anonymity. Besides, An's scheme lacks password change facility which is an important part of password-based user authentication schemes. We remove drawbacks from An's scheme by means of proposing an improved user authentication scheme. In addition, to resist various security threats, the proposed scheme incorporates features of password changing and user anonymity. The rest of this paper is arranged as follows. In Section 2, we review An's user authentication scheme.  Section 3 is about cryptanalysis of An's scheme. In Section 4, we present our improved scheme.  Section 5 is about security analysis of the improved scheme. In Section 6, we compare the improved scheme with related schemes. Finally, the conclusion is presented in Section 7. 

## 2. Review of An's Scheme

The notations useful in this paper are summarized along with their description in Table 1. In this section, we review An's scheme [27] which is an enhanced version of Das's scheme [26]. It has three phases: registration phase, login phase and authentication phase. Registration phase is carried over a secure channel whereas login phase, and authentication phase are carried over an insecure channel. There are three participants in the scheme, the user (*C*_*i*_), the server (*S*_*i*_), and the registration centre (*R*), where *R* is assumed to be a trusted party. Details of each phase are given in the following subsections.

### 2.1. Registration Phase

In the beginning of scheme, the registration centre *R* and the user *C*_*i*_ carry out this phase involving the following steps.

1. *C*_*i*_ submits his identity ID_*i*_ and information (PW_*i*_ ⊕ *K*_*i*_) containing password to *R* via a secure channel. *C*_*i*_ also submits information (*B*_*i*_ ⊕ *K*_*i*_) containing his biometrics via the specific device to *R*; here *K*_*i*_ is a random number chosen by *C*_*i*_.
2. *R*computes *f*_*i*_ = *h*(*B*_*i*_ ⊕ *K*_*i*_), *r*_*i*_ = *h*(PW_*i*_ ⊕ *K*_*i*_) ⊕ *f*_*i*_, and *e*_*i*_ = *h*(ID_*i*_||*x*_*s*_) ⊕ *r*_*i*_, where *x*_*s*_ is a secret key generated and maintained by *S*_*i*_. Then *R* stores {ID_*i*_, *f*_*i*_, *e*_*i*_, *h*(·)} in a smart card SC_*i*_ for user and provides it to *C*_*i*_ via a secure channel.
3. On receiving SC_*i*_ = {ID_*i*_, *f*_*i*_, *e*_*i*_, *h*(·)}, the user stores the random number *K*_*i*_ into SC_*i*_ issued by *R* so that now SC_*i*_ = {ID_*i*_, *f*_*i*_, *e*_*i*_, *h*(·)}.

### 2.2. Login Phase

When the user *C*_*i*_ wishes to login the server *S*_*i*_, the user and his smart card SC_*i*_ perform the following steps.

(1) *C*_*i*_ inserts his smart card into a card reader and inputs his biometrics information *B*_*i*_ on the specific device. SC_*i*_ computes *h*(*B*_*i*_ ⊕ *K*_*i*_) and verifies if *f*_*i*_ = *h*(*B*_*i*_ ⊕ *K*_*i*_) or not. If this biometrics information matches, *C*_*i*_ passes the biometrics verification.
(2) *C*_*i*_ inputs his ID_*i*_ and PW_*i*_; then SC_*i*_ generates a random number *R*_*c*_ and computes the following equations:
  (1) $$\begin{array}{c} r_{i}^{\prime }=h\left(\text{P}{\text{W}}_{i}⊕K_{i}\right)⊕f_{i},\\ M_{1}=e_{i}⊕r_{i}^{\prime },\\ M_{2}=M_{1}⊕R_{c},\\ M_{3}=h\left(M_{1}||R_{c}\right). \end{array}$$
(3) *C*_*i*_ sends the login request = {ID_*i*_, *M*_2_, *M*_3_} to *S*_*i*_.

### 2.3. Authentication Phase

On receiving the request login = {ID_*i*_, *M*_2_, *M*_3_} from *C*_*i*_, the server *S*_*i*_ and the user *C*_*i*_ perform the following steps to authenticate each other.

(1) *S*_*i*_ first checks the format of ID_*i*_. If ID_*i*_ is valid, *S*_*i*_ computes *M*_4_ = *h*(ID_*i*_||*x*_*s*_) and *M*_5_ = *M*_2_ ⊕ *M*_4_.
(2) *S*_*i*_ checks if *M*_3_ = *h*(*M*_4_||*M*_5_) or not. If both are equal, it generates a random number *R*_*s*_ and computes the following equations:
  (2) $$\begin{array}{c} M_{6}=M_{4}⊕R_{s},\\ M_{7}=h\left(M_{4}||R_{s}\right). \end{array}$$
  Then, *S*_*i*_ sends the reply message = {*M*_6_, *M*_7_} for its authentication to *C*_*i*_.
(3) On receiving {*M*_6_, *M*_7_} from *S*_*i*_, the user *C*_*i*_ computes *M*_8_ = *M*_6_ ⊕ *M*_1_ and checks if *M*_7_ = *h*(*M*_1_||*M*_8_) or not. If both are equal, *C*_*i*_ computes *M*_9_ = *h*(*M*_1_||*R*_*c*_||*M*_8_) and sends the reply message {*M*_9_} for its authentication to *S*_*i*_.
(4) On receiving {*M*_9_} from *C*_*i*_, the server checks if *M*_9_ = *h*(*M*_4_||*M*_5_||*R*_*s*_) or not. If both are equal, *S*_*i*_ accepts the login request = {ID_*i*_, *M*_2_, *M*_3_} of *C*_*i*_.

## 3. Cryptanalysis of An's Scheme 

This section is about security problems in An's scheme. Here we show that an attacker *U*_*a*_ can mount different types of attacks on the scheme. Independent researches by Kocher and Messerges [28, 29] show that it is possible to extract the values stored inside a smart card. So we assume that *U*_*a*_ can extract out parameters stored inside a user's smart card. 

### 3.1. Online Password Guessing Attack

If *U*_*a*_ obtains the smart card SC_*i*_ of user *C*_*i*_ and extracts [28, 29] the values {ID_*i*_, *f*_*i*_, *e*_*i*_, *K*_*i*_, *h*(·)} stored inside it, then he can mount online password guessing attack as explained below.

(1) *U*_*a*_ computes
  (3) ei⊕fi=[h(IDi||xs)⊕ri]⊕fi=[h(IDi||xs)⊕h(PWi⊕Ki)⊕fi]⊕fi=[h(IDi||xs)⊕h(PWi⊕Ki)]to  obtain  [h(IDi||xs)⊕h(PWi⊕Ki)].
(2) *U*_*a*_ guesses PW_*a*_ as user's possible password and computes *M*_1*a*_ = [*e*_*i*_ ⊕ *f*_*i*_] ⊕ *h*(PW_*a*_ ⊕ *K*_*i*_). Then *U*_*a*_ computes *M*_2*a*_ = *M*_1*a*_ ⊕ *R*_*ca*_ and *M*_3*a*_ = *h*(*M*_1*a*_||*R*_*ca*_), where *R*_*ca*_ is the random number generated by the system of *U*_*a*_. *He* sends {ID_*i*_, *M*_2*a*_, *M*_3*a*_} as login request to *S*_*i*_.
(3) If *U*_*a*_ does not receive any response from *S*_*i*_ then he repeats step (2) with some other guess for user's password. But if *U*_*a*_ receives response message from *S*_*i*_, then it implies that his guessed password PW_*a*_ is correct.

### 3.2. Offline Password Guessing Attack

In the scheme, *U*_*a*_ can easily identify the login request corresponding to a smart card since both contain the identity of user. If *U*_*a*_ extracts [28, 29] the values {ID_*i*_, *f*_*i*_, *e*_*i*_, *K*_*i*_, *h*(·)} from the smart card SC_*i*_ of user *C*_*i*_ and intercepts the login request = {ID_*i*_, *M*_2_, *M*_3_} from open network, then he can mount offline password guessing attack as explained below.

(1) *U*_*a*_ computes
  (4) ei⊕fi=[h(IDi||xs)⊕ri]⊕fi=[h(IDi||xs)⊕h(PWi⊕Ki)⊕fi]⊕fi=[h(IDi||xs)⊕h(PWi⊕Ki)]to  obtain  [h(IDi||xs)⊕h(PWi⊕Ki)].
(2) *U*_*a*_ guesses PW_*a*_ as user's possible password and computes *M*_1*a*_ = [*e*_*i*_ ⊕ *f*_*i*_] ⊕ *h*(PW_*a*_ ⊕ *K*_*i*_).
(3) *U*_*a*_ computes *R*_*ca*_ = *M*_2_ ⊕ *M*_1*a*_ and *M*_3*a*_ = *h*(*M*_1*a*_||*R*_*ca*_), and finally compares *M*_3*a*_ with *M*_3_. For *M*_3*a*_ ≠ *M*_3_, he repeats from step (2) with some other guess for user's password. But if *M*_3*a*_ = *M*_3_, then it provides *U*_*a*_ with the exact password PW_*i*_ of *C*_*i*_.

### 3.3. User Impersonation Attack

As just discussed in previous subsections, *U*_*a*_ can guess a user's password if he obtains the smart card of user. It is noticeable that the successful process of password guessing (online or offline manner) also yields *M*_1*a*_ = *h*(ID_*i*_||*x*_*s*_). In fact, *h*(ID_*i*_||*x*_*s*_) is the key value required to compute a valid login request or valid reply messages. Further, *U*_*a*_ has easy access to user's identity ID_*i*_ from SC_*i*_ = {ID_*i*_, *f*_*i*_, *e*_*i*_, *K*_*i*_, *h*(·)} or from the login request = {ID_*i*_, *M*_2_, *M*_3_} of *C*_*i*_. Having *h*(ID_*i*_||*x*_*s*_) and ID_*i*_ in hand, *U*_*a*_ can impersonate the user *C*_*i*_ as explained below.

(1) *U*_*a*_ generates a random number *R*_*ca*_ in his system and computes
  (5) M2a=M1a⊕Rca,M3a=h(M1a||Rca).
  Then *U*_*a*_ sends the login request = {ID_*i*_, *M*_2*a*_, *M*_3*a*_} to *S*_*i*_.
(2) On receiving {ID_*i*_, *M*_2*a*_, *M*_3*a*_}, the server *S*_*i*_ first checks the format of ID_*i*_. Clearly, *S*_*i*_ would proceed further because ID_*i*_ is the identity of a legitimate registered user and hence it is in valid format.
(3) *S*_*i*_ computes *M*_4_ = *h*(ID_*i*_||*x*_*s*_) and *M*_5_ = *M*_2*a*_ ⊕ *M*_4_ and checks if *M*_3*a*_ = *h*(*M*_4_||*M*_5_); clearly it would hold. Therefore *S*_*i*_ believes that the login request = {ID_*i*_, *M*_2*a*_, *M*_3*a*_} is from the legitimate user.
(4) *S*_*i*_ generates a random number *R*_*s*_ and computes *M*_6_ = *M*_4_ ⊕ *R*_*s*_ and *M*_7_ = *h*(*M*_4_||*R*_*s*_). Then *S*_*i*_ transmits the reply message {*M*_6_, *M*_7_}.
(5) On receiving {*M*_6_, *M*_7_} from *S*_*i*_, the attacker *U*_*a*_ first obtains the random number *R*_*s*_ by computing *M*_8*a*_ = *M*_6_ ⊕ *M*_1*a*_. Next, it computes *M*_9*a*_ = *h*(*M*_1*a*_||*R*_*ca*_||*M*_8_) and sends {*M*_9*a*_} to *S*_*i*_.
(6) On receiving {*M*_9_}, the server *S*_*i*_ checks if *M*_9_ = *h*(*M*_4_||*M*_5_||*R*_*s*_) or not. Clearly, this would hold, so *S*_*i*_ will accept the login request = {ID_*i*_, *M*_2*a*_, *M*_3*a*_}.

### 3.4. Server Impersonation Attack

*U*_*a*_ can easily impersonate the legal server *S*_*i*_ to cheat the user *C*_*i*_ whose information {ID_*i*_  and  *M*_1*a*_ = *h*(ID_*i*_||*x*_*s*_)} he possesses as described in Section 3.3. To masquerade as *S*_*i*_ the attacker proceeds in the following manner.

1. *U*_*a*_ can easily recognize the login request = {ID_*i*_, *M*_2_, *M*_3_} of *C*_*i*_ transmitted over open channel as he possesses the identity ID_*i*_ of *C*_*i*_. So when *C*_*i*_ sends his login request = {ID_*i*_, *M*_2_, *M*_3_} to *S*_*i*_, the attacker *U*_*a*_ intercepts and blocks it from reaching *S*_*i*_.
2. *U*_*a*_ first obtains the random number *R*_*c*_ by computing *M*_5*a*_ = *M*_2_ ⊕ *M*_1*a*_. Next, he generates a random number *R*_*sa*_ in his system and computes *M*_6*a*_ = *M*_1*a*_ ⊕ *R*_*sa*_ and *M*_7*a*_ = *h*(*M*_1*a*_||*R*_*sa*_). Then *U*_*a*_ transmits the reply message {*M*_6*a*_, *M*_7*a*_} to *C*_*i*_.
3. On receiving {*M*_6*a*_, *M*_7*a*_}, the user *C*_*i*_ first obtains the random number *R*_*sa*_ by computing *M*_8_ = *M*_6*a*_ ⊕ *M*_1_, where *M*_1_ = *h*(ID_*i*_||*x*_*s*_). Next, he checks if *M*_7*a*_ = *h*(*M*_1_||*M*_8_) or not. Clearly, this equivalence will hold and hence *C*_*i*_ will believe that he is communicating with the intended server. However, it is the clever attacker *U*_*a*_ who is deceiving *C*_*i*_.

### 3.5. Lack of Mutual Authentication

Like Das's scheme [26], the enhanced scheme by An also fails to resist user impersonation attack and server impersonation attack as described in Sections 3.3 and 3.4. In fact, if  *U*_*a*_ extracts values {ID_*i*_, *f*_*i*_, *e*_*i*_, *K*_*i*_, *h*(·)} from the smart card SC_*i*_ of user *C*_*i*_ and successfully obtains the secret value *h*(ID_*i*_||*x*_*s*_), then he can easily craft valid login request and reply messages so as to deceive the legal user or the legal server. Therefore, the scheme loses mutual authentication feature.

### 3.6. Lack of User Anonymity

In An's scheme, *C*_*i*_ sends {ID_*i*_, *M*_2_, *M*_3_} as his login request to *S*_*i*_ through an insecure channel. User's identity ID_*i*_ is openly available if an attacker *U*_*a*_ intercepts the login request of *C*_*i*_ from the open channel. Moreover, identity ID_*i*_ is also stored inside user's smart card SC_*i*_. Having ID_*i*_ in hand, it is easy for *U*_*a*_ to craft threats against *C*_*i*_. To the worst, *U*_*a*_ may be able to compromise user's biometrics information which would result in serious consequences. Thus, the scheme does not provide user anonymity.

## 4. The Proposed Scheme

**Figure 1:**
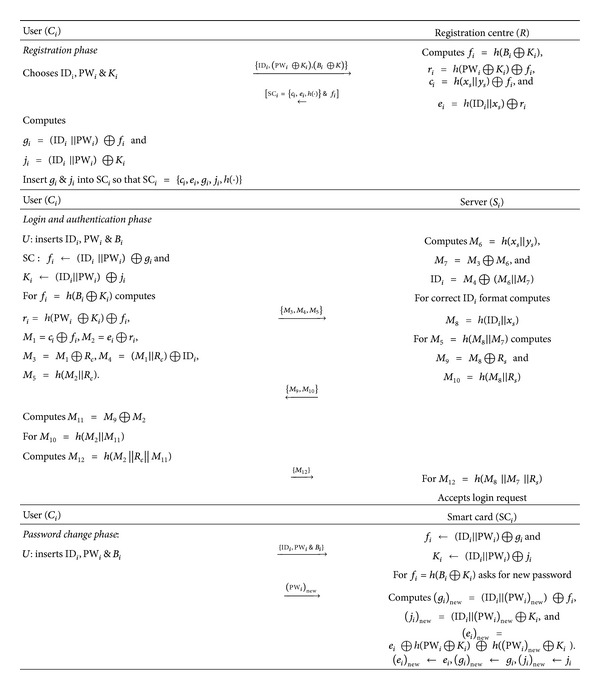
The proposed scheme.

In this section, we propose a new user authentication scheme which is an improvement of An's scheme. In addition to resist the security problems found in An's scheme, it also provides password change phase with which user can change his password at his will. It has four phases: registration phase, login phase, authentication phase and password change phase. Registration phase, and password change phase are carried over a secure channel whereas login phase and authentication phase are carried over an insecure channel. It also consists of three participants, the user (*C*_*i*_), the server (*S*_*i*_), and the registration centre (*R*). In the proposed scheme, the server maintains two secret keys *x*_*s*_ and *y*_*s*_. Details of each phase along with Figure 1 are given in the following.

### 4.1. Registration Phase

Before starting the scheme, the registration centre *R* and the user *C*_*i*_ carry out this phase involving the following steps.

(1) *C*_*i*_ submits his identity ID_*i*_ and information (PW_*i*_ ⊕ *K*_*i*_) containing password to *R* via a secure channel. *C*_*i*_ also submits information (*B*_*i*_ ⊕ *K*_*i*_) containing his biometrics via a specific device to *R*; here *K*_*i*_ is a random number chosen by *C*_*i*_.
(2) *R* computes the following values:
  (6) $$\begin{array}{c} f_{i}=h\left(B_{i}⊕K_{i}\right),\\ r_{i}=h\left({\text{PW}}_{i}⊕K_{i}\right)⊕f_{i},\\ c_{i}=h\left(x_{s}||y_{s}\right)⊕f_{i},\\ e_{i}=h\left({\text{ID}}_{i}||x_{s}\right)⊕r_{i}, \end{array}$$
  where *R* stores {*c*_*i*_, *e*_*i*_, *h*(·)} in a smart card SC_*i*_ for user. Then *R* provides SC_*i*_ = {*c*_*i*_, *e*_*i*_, *h*(·)} and *f*_*i*_ to the user *C*_*i*_ via a secure channel.
(3) On receiving [SC_*i*_ = {*c*_*i*_, *e*_*i*_, *h*(·)}  &  *f*_*i*_], the user computes the following values:
  (7) $$\begin{array}{c} g_{i}=\left({\text{ID}}_{i}||{\text{PW}}_{i}\right)⊕f_{i},\\ j_{i}=\left({\text{ID}}_{i}||{\text{PW}}_{i}\right)⊕K_{i}, \end{array}$$
  where *C*_*i*_ inserts *g*_*i*_ and *j*_*i*_ into SC_*i*_ issued by *R* so that now SC_*i*_ = {*c*_*i*_, *e*_*i*_, *g*_*i*_, *j*_*i*_, *h*(·)}.

### 4.2. Login Phase

When the user *C*_*i*_ wishes to login the server *S*_*i*_, the user and his smart card SC_*i*_ perform the following steps.

(1) *C*_*i*_ inserts his smart card into a card reader, keys in his identity ID_*i*_, and password PW_*i*_ and inputs his biometrics information *B*_*i*_ on the specific device.
(2) SC_*i*_ retrieves *f*_*i*_ ← (ID_*i*_||PW_*i*_) ⊕ *g*_*i*_ and *K*_*i*_ ← (ID_*i*_||PW_*i*_) ⊕ *j*_*i*_. It then checks if *f*_*i*_ = *h*(*B*_*i*_ ⊕ *K*_*i*_) or not. If this biometrics information matches, *C*_*i*_ passes the biometrics verification; otherwise SC_*i*_ terminates the sesion. This process also verifies the correctness of inserted ID_*i*_ and PW_*i*_.
(3) SC_*i*_ generates a random number *R*_*c*_ and computes the following equations:
  (8) ri=h(PWi⊕Ki)⊕fi,M1=ci⊕fi (which  is  indeed  h(xs||ys)),M2=ei⊕ri (which  is  indeed  h(IDi||xs)),M3=M1⊕Rc (which  is  indeed  h(xs||ys)⊕Rc),M4=(M1||Rc)⊕IDi(which  is  indeed  [(h(xs||ys)||Rc)⊕IDi]),M5=h(M2||Rc),(which  is  indeed  h(h(IDi||xs)||Rc)).
(4) *C*_*i*_ sends the login request = {*M*_3_, *M*_4_, *M*_5_} to *S*_*i*_.

### 4.3. Authentication Phase

On receiving the request login = {*M*_3_, *M*_4_, *M*_5_} from *C*_*i*_, the server *S*_*i*_ and the user *C*_*i*_ perform the following steps to authenticate each other.

(1) *S*_*i*_ computes the following values:
  (9) $$\begin{array}{c} M_{6}=h\left(x_{s}||y_{s}\right),\\ M_{7}=M_{3}⊕M_{6}\;\left(\text{which}\;\text{is}\;\text{indeed}\;R_{c}\right),\\ {\text{ID}}_{i}=M_{4}⊕\left(M_{6}||M_{7}\right). \end{array}$$
(2) *S*_*i*_ checks the format of ID_*i*_. If ID_*i*_ is valid, *S*_*i*_ computes *M*_8_ = *h*(ID_*i*_||*x*_*s*_). It then checks if *M*_5_ = *h*(*M*_8_||*M*_7_). If both are equal, *S*_*i*_ generates a random number *R*_*s*_ and computes:
  (10) M9=M8⊕Rs(which  is  indeed  h(IDi||xs)⊕Rs)M10=h(M8||Rs)(which  is  indeed  h(h(IDi||xs)||Rs)).
  Then, *S*_*i*_ sends the reply message = {*M*_9_, *M*_10_} for its authentication to *C*_*i*_.
(3) On receiving {*M*_9_, *M*_10_} from *S*_*i*_, the user *C*_*i*_ computes *M*_11_ = *M*_9_ ⊕ *M*_2_ (which is indeed *R*_*s*_). It then checks if *M*_10_ = *h*(*M*_2_||*M*_11_) or not. If both are equal, *C*_*i*_ computes *M*_12_ = *h*(*M*_2_||*R*_*c*_||*M*_11_) (which is indeed *h*[*h*(ID_*i*_||*x*_*s*_)||*R*_*c*_||*R*_*s*_]). Then *C*_*i*_ sends the reply message {*M*_12_} for its authentication to *S*_*i*_.
(4) On receiving {*M*_12_} from *C*_*i*_, the server checks if *M*_12_ = *h*(*M*_8_||*M*_7_||*R*_*s*_) or not. If both are equal, *S*_*i*_ accepts the login request = {*M*_3_, *M*_4_, *M*_5_} of *C*_*i*_.

### 4.4. Password Change Phase

When the user wishes to change his old password PW_*i*_, he invokes this phase. Details of the steps required to update the smart card SC_*i*_ with new password (PW_*i*_)_new_ are as follows.

(1) *C*_*i*_ inserts his smart card into a card reader, keys in his identity ID_*i*_, and password PW_*i*_ and inputs his biometrics information *B*_*i*_ on the specific device.
(2) SC_*i*_ retrieves *f*_*i*_ ← (ID_*i*_||PW_*i*_) ⊕ *g*_*i*_ and *K*_*i*_ ← (ID_*i*_||PW_*i*_) ⊕ *j*_*i*_. It then checks if *f*_*i*_ = *h*(*B*_*i*_ ⊕ *K*_*i*_) or not. If this biometrics information matches, *C*_*i*_ passes the biometrics verification, otherwise terminates the session. This process also verifies the correctness of inserted ID_*i*_ and PW_*i*_. Then SC_*i*_ allows the user to enter the new password (PW_*i*_)_new_.
(3) SC_*i*_ computes the following equations:
  (11) (gi)new=(IDi||(PWi)new)⊕fi,(ji)new=(IDi||(PWi)new)⊕Ki,(ei)new=ei⊕h(PWi⊕Ki)⊕h((PWi)new⊕Ki).
(4) SC_*i*_ replaces *e*_*i*_, *g*_*i*_, and *j*_*i*_ with (*e*_*i*_)_new_, (*g*_*i*_)_new_ and (*j*_*i*_)_new_, respectively.

## 5. Security Analysis of the Proposed Scheme

In this section, we analyze security of the proposed scheme. We show that the scheme remains unaffected even if an attacker *U*_*a*_ extracts [28, 29] all the values stored inside a user's smart card. 

### 5.1. Online Password Guessing Attack

On having access to user's smart card SC_*i*_ an attacker *U*_*a*_ can extract [28, 29] all values {*c*_*i*_, *e*_*i*_, *g*_*i*_, *j*_*i*_, *h*(·)} from it. In order to compute *e*_*i*_ ⊕ *f*_*i*_ and obtain [*h*(ID_*i*_||*x*_*s*_) ⊕ *h*(PW_*i*_ ⊕ *K*_*i*_)], he requires *f*_*i*_. But *U*_*a*_ cannot obtain *f*_*i*_ from *g*_*i*_ = (ID_*i*_||PW_*i*_) ⊕ *f*_*i*_ as he does not know about user's identity ID_*i*_ and password PW_*i*_. The attacker *U*_*a*_ can obtain *f*_*i*_ ⊕ *K*_*i*_ by performing *g*_*i*_ ⊕ *j*_*i*_ = [(ID_*i*_||PW_*i*_) ⊕ *f*_*i*_]⊕[(ID_*i*_||PW_*i*_) ⊕ *K*_*i*_]. Next, he can compute

(12) ei⊕(fi⊕Ki) =[h(IDi||xs)⊕ri]⊕(fi⊕Ki) =[h(IDi||xs)⊕h(PWi⊕Ki)⊕fi]⊕(fi⊕Ki) =h(IDi||xs)⊕h(PWi⊕Ki)⊕Ki.

But *U*_*a*_ cannot compute forged *M*_2*a*_  ( = *h*(ID_*i*_||*x*_*s*_)) = [*e*_*i*_ ⊕ *f*_*i*_ ⊕ *K*_*i*_] ⊕ *h*(PW_*a*_ ⊕ *K*_*i*_) using a guessed password PW_*a*_ because it requires knowledge of *K*_*i*_. It is troublesome for *U*_*a*_ to obtain *K*_*i*_ because *K*_*i*_ is not stored in plaintext inside user's smart card but is stored securely in *j*_*i*_ = (ID_*i*_||PW_*i*_) ⊕ *K*_*i*_. Further *U*_*a*_ cannot obtain *K*_*i*_ from *j*_*i*_ without knowing ID_*i*_ and password PW_*i*_. Besides, *U*_*a*_ cannot compute *M*_1*a*_  ( = *h*(*x*_*s*_||*y*_*s*_)) = (*c*_*i*_ ⊕ *f*_*i*_) as he does not have access to *f*_*i*_. Moreover, *U*_*a*_ does not have ID_*i*_ of *C*_*i*_ as ID_*i*_ is not stored in plaintext inside user's smart card. Thus, *U*_*a*_ cannot compute a login request {*M*_3*a*_, *M*_4*a*_, *M*_5*a*_} in a way so as to guess user's password in an online manner. Hence, the proposed scheme withstands online password guessing attack.

### 5.2. Offline Password Guessing Attack

Suppose *U*_*a*_ obtains the smart card of some user. Though *U*_*a*_ can intercept login message of any user from open channel, he cannot relate a user's smart card with its corresponding login request. This is due to the fact that, unlike An's scheme, in the proposed scheme user's identity in plaintext is neither stored inside user's smart card nor transmitted in login request. As a result, *U*_*a*_ cannot combine values extracted from a user's smart card with values of corresponding login request to guess user's password in an offline manner. If we consider the situation that *U*_*a*_ somehow happens to get the correct combination of user's smart card and login request, we show that still *U*_*a*_ cannot mount offline password guessing attack. To guess password of *C*_*i*_ and then verify the guess, *U*_*a*_ can use *M*_5_ = *h*(*M*_2_||*R*_*c*_) provided that he possesses the values {[*h*(ID_*i*_||*x*_*s*_) ⊕ *h*(PW_*i*_ ⊕ *K*_*i*_) ⊕ *K*_*i*_], *K*_*i*_  and  *R*_*c*_} in hand. As explained in Section 5.1, *U*_*a*_ can obtain [*h*(ID_*i*_||*x*_*s*_) ⊕ *h*(PW_*i*_ ⊕ *K*_*i*_) ⊕ *K*_*i*_] using {*g*_*i*_, *j*_*i*_  and  *e*_*i*_} extracted [28, 29] from SC_*i*_, but he cannot obtain the random number *K*_*i*_. Besides, *U*_*a*_ cannot obtain the random number *R*_*c*_ using *M*_3_ = *M*_1_ ⊕ *R*_*c*_ without having *M*_1_  ( = *h*(*x*_*s*_||*y*_*s*_)) and *U*_*a*_ fails to obtain *M*_1_  ( = *h*(*x*_*s*_||*y*_*s*_)) as discussed in Section 5.1. Thus an attacker *U*_*a*_ cannot guess user's password in an offline manner. 

### 5.3. User Impersonation and Server Impersonation Attack

To impersonate a legal user, *U*_*a*_ should possess *M*_1_ = *h*(*x*_*s*_||*y*_*s*_) and *M*_2_ = *h*(ID_*i*_||*x*_*s*_); otherwise he cannot compute a valid login request {*M*_3*a*_, *M*_4*a*_, *M*_5*a*_} or a valid reply message {*M*_12*a*_}. The value *h*(ID_*i*_||*x*_*s*_) is equally important if *U*_*a*_ wishes to masquerade as legal server. Unlike An's scheme, in the proposed scheme *U*_*a*_ is not able to obtain *M*_2_  ( = *M*_8_) = *h*(ID_*i*_||*x*_*s*_) while making attempts of guessing user's password. This is due to the fact that password guessing is not feasible as explained in Sections 5.1 and 5.2. Moreover, *U*_*a*_ cannot obtain *M*_1_ = *h*(*x*_*s*_||*y*_*s*_) (i) from *M*_3_ = *M*_1_ ⊕ *R*_*c*_ obtained by intercepting the login request of *C*_*i*_ because of not having random number *R*_*c*_ and (ii) from *c*_*i*_ = *h*(*x*_*s*_||*y*_*s*_) ⊕ *f*_*i*_ extracted from user's smart card without knowing *f*_*i*_. Thus, the proposed scheme resists impersonation attacks. 

### 5.4. Supporting Mutual Authentication

The success of mutual authentication in the proposed scheme follows directly from resistance against user impersonation attack and server impersonation attack as described in Section 5.3. In fact, *U*_*a*_ has many hurdles before him to act as a legal user or a legal server: (i) the secret keys *x*_*s*_ and *y*_*s*_ maintained by the server are unknown for *U*_*a*_ and (ii) *U*_*a*_ has no access to the identity ID_*i*_ of user *C*_*i*_. As a result, *U*_*a*_ cannot compute *h*(*x*_*s*_||*y*_*s*_) and *h*(ID_*i*_||*x*_*s*_) required to mount impersonation attacks. Besides, *U*_*a*_ has no method to retrieve these values either from the parameters extracted out of user's smart card or from the login request or using both. Therefore, the proposed scheme provides proper mutual authentication. 

### 5.5. Providing User Anonymity and User Untraceability

In the proposed scheme, user's plaintext identity ID_*i*_ is completely out of scene; it is neither stored in user's smart card SC_*i*_ nor sent in any of the login-authentication messages transmitted over insecure network. If *U*_*a*_ extracts [28, 29] the values {*c*_*i*_, *e*_*i*_, *g*_*i*_, *j*_*i*_, *h*(·)} from SC_*i*_, we explain in the following that he cannot obtain ID_*i*_ of *C*_*i*_. To guess ID_*i*_ from *g*_*i*_ = (ID_*i*_||PW_*i*_) ⊕ *f*_*i*_ and from *j*_*i*_ = (ID_*i*_||PW_*i*_) ⊕ *K*_*i*_, the attacker must have the knowledge of {PW_*i*_, *f*_*i*_} and {PW_*i*_, *K*_*i*_}, respectively. *U*_*a*_ cannot guess out ID_*i*_ from *e*_*i*_ = *h*(ID_*i*_||*x*_*s*_) ⊕ *r*_*i*_ without knowing *r*_*i*_ and *x*_*s*_. If *U*_*a*_ intercepts a login request {*M*_3_, *M*_4_, *M*_5_} or the reply message {*M*_9_, *M*_10_}/{*M*_12_}, he cannot guess out ID_*i*_ using {*M*_5_, *M*_10_, *M*_12_} without the knowledge of {*x*_*s*_, *R*_*c*_  and  *R*_*s*_}. Besides, it is not feasible for *U*_*a*_ to retrieve ID_*i*_ out of {*e*_*i*_, *M*_5_, *M*_10_, *M*_12_} due to one-way property of hash function. Moreover, each value {*M*_3_, *M*_4_, *M*_5_, *M*_9_, *M*_10_, *M*_12_} transmitted over insecure network is dynamic in nature by virtue of random numbers *R*_*c*_ and *R*_*s*_ which are different for each session. Thus, *U*_*a*_ can neither obtain user's identity ID_*i*_ nor can he trace the legal user by means of observing and analyzing some fixed parameter in the login request or the reply messages. Hence, the scheme provides user anonymity as well as user untraceability.

### 5.6. Providing Password Change Facility

In An's scheme, once user chooses his password during registration phase, it is fixed forever as user cannot change his password at his will. Probably the author might have opined that in the presence of biometrics verification procedure there is no need of password change facility. Undoubtedly, it is very difficult to forge copy or compromise biometrics, but once compromised then biometrics cannot be changed like passwords. So we opine that if password is employed in user authentication scheme then there should be the provision to facilitate the user to freely change his password. The proposed scheme provides password changing facility with which a user can freely (without interacting with server) change his old password to a new one whenever he feels to do so. Before updating stored values with the new password (PW_*i*_)_new_, the smart card verifies the correctness of identity ID_*i*_ old password PW_*i*_ along with verifying the biometrics information *f*_*i*_ = *h*(*B*_*i*_ ⊕ *K*_*i*_). Thus the proposed scheme provides secure and easy password changing facility.

## 6. Comparison

In this section, we examine the proposed scheme by means of comparing its efficiency with Li-Hwang's scheme [19], Das's scheme [26], and An's scheme [27].  Table 2 displays comparison of security attributes and Table 3 displays comparison of computational load in terms of hash functions. Comparison in Table 2 shows that the proposed scheme resists various attacks possible on schemes [19, 26, 27] and provides additional feature of user anonymity with untraceability. Besides, it also restores password change facility which is provided by original versions [19, 26] but is missing in An's scheme [27]. As Table 3 shows, the proposed scheme carries only two additional hash operations over its immediate predecessor scheme [27]. The important aspect about the proposed scheme is minor increase of two hash functions in computational load to achieve higher efficiency as compared to other schemes [19, 26, 27].

## 7. Conclusion

This paper shows that the recently proposed biometrics-based user authentication scheme by An is susceptible to many threats. Once an attacker obtains the smart card of a legal user, he can guess user's password and impersonate the user. Further, the attacker can also cheat the user by masquerading as the legal server. Consequently, the scheme fails to provide mutual authentication. Besides, the scheme also suffers from the restriction of static password. We have proposed a new scheme based on the design of An's scheme so as to fix the problems identified in An's scheme. In the proposed scheme an attacker cannot figure out the identity of user either from the smart card or by intercepting all login-authentication messages transmitted over insecure network. Analysis and comparison show improved performance of the proposed scheme.

## Figures and Tables

**Table 1:** Notations with their description.

Notations	Description
*R*	Trusted registration centre
*S*_*i*_	Server
*C*_*i*_	User
ID_*i*_	Identity of *C*_*i*_
PW_*i*_	Password of *C*_*i*_
*B*_*i*_	Biometric template of *C*_*i*_
SC_*i*_	Smart card of *C*_*i*_
*K*_*i*_	Random number chosen by *C*_*i*_
*R*_*c*_	Random number generated by SC_*i*_ of *C*_*i*_
*R*_*s*_	Random number generated by *S*_*i*_
*U*_*a*_	Attacker
*x*_*s*_ and *y*_*s*_	Secret keys maintained by *S*_*i*_
*h*(·)	One-way hash function
⊕	Bitwise XOR operator
||	Concatenation operator

**Table 2:** Comparison of security attributes.

Security attributes	Schemes			
	Li-Hwang's [19]	Das's [26]	An's [27]	Ours
Resist online PW_*i*_ guessing attack	No	No	No	Yes
Resist offline PW_*i*_ guessing attack	No	No	No	Yes
Resist user impersonation attack	No	No	No	Yes
Resist server impersonation attack	No	No	No	Yes
Provides mutual authentication	No	No	No	Yes
Provides PW_*i*_ * *change facility	Yes	Yes	No	Yes
Provides user anonymity	No	No	No	Yes

**Table 3:** Comparison of computational load in terms of hash functions.

Phases	Schemes			
	Li-Hwang's [19]	Das's [26]	An's [27]	Ours
Registration phase	3 *h*(·)	3 *h*(·)	3 *h*(·)	4 *h*(·)
Login phase	2 *h*(·)	2 *h*(·)	3 *h*(·)	3 *h*(·)
Authentication phase	5 *h*(·)	8 *h*(·)	6 *h*(·)	7 *h*(·)

Total	10 *h*(·)	13 *h*(·)	12 *h*(·)	14 *h*(·)
